# Exercise training improves vascular reactivity in ovariectomized rats subjected to myocardial infarction

**DOI:** 10.1371/journal.pone.0215568

**Published:** 2019-04-24

**Authors:** Suelen Guedes de Oliveira, Erick Roberto Gonçalves Claudio, Simone Alves de Almeida, Vinicius Mengal, Fabricio Bragança da Silva, Nyam Florêncio Silva, Helder Mauad, Glaucia Rodrigues de Abreu

**Affiliations:** 1 Department of Physiological Sciences, Health Sciences Center, Federal University of Espírito Santo, Vitória-ES, Brazil; 2 Department of Morphology, Health Sciences Center, Federal University of Espírito Santo, Vitória-ES, Brazil; Max Delbruck Centrum fur Molekulare Medizin Berlin Buch, GERMANY

## Abstract

The aim of this study was to evaluate the effects of exercise training (ET) on the aortic vascular reactivity of ovariectomized and infarcted rats. The animals were divided into 5 groups: Control, Ovariectomized + SHAM sedentary (OVX+SHAM_SED_), OVX+SHAM and ET (OVX+SHAM_ET_), OVX + Myocardial Infarction sedentary (OVX+MI_SED_), and OVX + MI and ET (OVX+MI_ET_). ET protocol (60 minutes/day, 5x/week) in a motorized treadmill began 15 days after MI and lasted 8 weeks. The endothelium-dependent and endothelium-independent vascular reactivity were evaluated as well as the role of the reactive oxygen species (ROS). Superoxide and nitric oxide (NO) production were analyzed *in situ* using DHE and DAF-2 fluorescence, respectively. The expression of gp91phox and of the antioxidant enzymes were evaluated by western blotting in the thoracic aorta samples. MI promoted a significant increase in the contractile response and impaired endothelium-mediated relaxation. However, ET prevented the impairment in the vascular reactivity in MI animals. In addition, the protein expression of gp91phox and superoxide production increased and the NO production decreased in the OVX+MI_SED_ group but not in the OVX+MI_ET_ group. Therefore, ET improves vascular reactivity in MI ovariectomized rats by preventing the increase in the expression of gp91phox and the decrease in the antioxidant enzymes, resulting in a normal ROS and NO production. Thus, ET can be an effective therapeutic strategy for improving the MI-induced vascular alterations in estrogen deficiency condition.

## Introduction

Estrogens play important functions in female organisms, such as the development of sexual organs, cellular proliferation, and bone maturation as well as in the protection of the cardiovascular system [1]. Women in the postmenopausal period become more susceptible to the development of cardiovascular diseases (CVD) such as the myocardial infarction (MI) which stands out because of its high incidence [1].

The cardiovascular remodeling after MI causes alterations in the structure and functioning of the myocardium as well as in the blood vessels. The impairments seem to be intensified in women after menopause because of the estrogen deficiency, mainly 17-β-estradiol (E2) [2], which worsens the autonomic regulation [3], vascular contractility [4] and relaxation because of the reduction in the nitric oxide (NO) bioavailability [5]. Subjects affected by MI shows an increase in vascular oxidative stress [6–9], augmenting the bind of NO with superoxide forming peroxynitrite (ONOO^-^) [10,11] and, therefore, leading to an endothelial dysfunction framework.

The regular practice of exercise training (ET) seems to be effective as a non-pharmacological treatment for a wide variety of conditions, including the cardiovascular alterations mediated by MI [12,13]. However, little is known about the effects of ET on vascular function in infarcted animals in the absence of ovarian hormones.

Several studies have shown that ET is able to counteract the oxidative stress mainly due to its antioxidant effects. Therefore, the improvement in the antioxidant system constitutes one of the major effects resulting from ET [14,15], as well as the modulation of the components that regulate the NADPH oxidase complex [16,17]

Accordingly, the hypothesis of the present study is that ET can prevent the changes in vascular function promoted by the MI, even with the estrogen deficiency. Thus, the aim of our study is to evaluate the effects of ET on the vascular reactivity in aortic rings of ovariectomized and infarcted rats as well as the role of reactive oxygen species (ROS).

## Material and methods

### Animals

Ten-week-old Wistar female rats (*Rattus Norvegicus Albinus*) with weights ranging from 200–230 grams were used. A total number of 60 rats was used in this study. The animals were randomly divided into 5 groups: Control, Ovariectomized + SHAM sedentary (OVX+SHAM_SED_), OVX+SHAM and ET (OVX+SHAM_ET_), OVX + Myocardial Infarction sedentary (OVX+MI_SED_), and OVX + MI and ET (OVX+MI_ET_). The animals were obtained from the university facility and were placed in collective cages with free access to water and food. The temperature was kept between 22 °C and 24 °C with a 12-hour light/dark cycle. The study was approved by the institutional ethical committee (Comissão de Ética no Uso de Animais–CEUA/UFES), under the protocol number 059/2012, and all experimental procedures were conducted according to the “Guide for the Care and Use of Laboratory Animals”.

### Ovariectomy

Animals were anesthetized with ketamine (50 mg/kg, Agener, Brazil) and xylazine (10 mg/kg, Bayer, Brazil). A 1.5-cm incision was made in the skin between the last costal ridge and thigh, followed by an incision in the muscle layer. The ovary was exteriorized and removed by ligating the uterine tube on both sides and then a suture was performed on the muscle and the skin. After surgery, animals received an antibiotic injection (Enrofloxacin—2.5% - 0.1 mL–*im*).

### MI induction

Seven days after OVX, a trichotomy was performed in the chest region in animals previously anesthetized with ketamine (50 mg/kg, Agener, Brazil) and xylazine (10 mg/kg, Bayer, Brazil). After asepsis, an incision was made in the skin, and the intercostal muscles were dissected. A suture in the form of a pouch was prepared for the rapid closure of the surgical incision after coronary ligation. A lateral incision at the level of the fourth intercostal space was performed to expose the heart, which was externalized, and the left anterior descending coronary artery was permanently occluded using 6.0 mono-nylon thread and a non-traumatic needle (Medical Line). After occlusion, the chest was immediately closed. The OVX+SHAM_ET_ and OVX+SHAM_SED_ groups were subjected to a sham surgical procedure, without occlusion of the artery. After the surgical procedure, the animals received antibiotic (Enrofloxacin—2.5%—0.1 mL—*im*).

### Exercise training protocol

Running training was performed on an electric treadmill (EP 131, Insight, Brazil), beginning 2 weeks after the MI induction. The protocol used was adapted from a previous study of our group [16]. Briefly, the OVX+MI_ET_ and OVX+SHAM_ET_ groups underwent 8 weeks of ET, for a duration of 60 minutes at a frequency of 5 times per week. The ET protocol was conducted during the same period of the day (between 4:00–6:00 p.m). The first week consisted of an adaptation period, where the animals started the training with the intensity of 3 meters/minute and duration of 10 minutes. The duration of the training was progressively increased over the days until on day 5 the animals reached the duration of 60 minutes at the same initial speed. The speed was gradually increased every 2 weeks to 3 m/min, 6 m/min, 9 m/min, and finally to 12 m/min.

### Tissue collection, weighing, and storage

After anesthesia with ketamine (50 mg/kg, Agener, Brazil) and xylazine (10 mg/kg, Bayer, Brazil), the rats were sacrificed by decapitation. The animals of the control group were euthanized in the proestrous phase of the estrous cycle when the levels of ovarian hormones are high, verified by the vaginal smear. After the main procedures, the following tissues were collected and weighed: retroperitoneal fat, parametrial fat, mesenteric fat, the uterus, and the lung, which was weighed wet and dry. To obtain the dry weight, the lung was placed in an oven heated at 98 °C for 24 hours. The heart was also collected and stored in formaldehyde, and segments of the aortic arteries were stored in a freezer at -80 °C for further analysis.

### Vascular reactivity

After the sacrifice, the chest of the animals was opened, and the descending thoracic aorta artery was removed and immersed in a modified Krebs solution composed of (in mM) NaCl 118; KCl 4.7; MgSO_4_ 1.2; CaCl_2_ 1.6; K_2_HPO_4_ 1.2; NaHCO_3_ 25; and glucose 5.5 and aerated with a carbogenic mixture containing 5% CO_2_ and 95% O_2_. The connective and adipose tissues were removed, and the artery was divided into sections of approximately 3.5 mm in length. Each vascular ring was placed in organ baths containing 5 mL of Krebs-Henseleit solution heated to 36 ± 0.5 °C and continuously gassed with the carbogenic mixture. Two stainless steel wires were passed through the lumen of the segments by positioning the wires parallel in the vessel lumen. One of the wires was attached to the bath wall and the other was connected vertically to an isometric voltage transducer. Changes in vessel diameter were recorded by a force transducer connected to a data acquisition system (AdInstruments, Australia). After mounted, the aortic rings were subjected to a resting tension of approximately 1 gram and readjusted when necessary during the 45-minute stabilization period. The rings were then contracted with potassium chloride (KCl; 75 mM concentration) until reproducible responses were obtained. After being washed again and when the basal values became stable, the rings were contracted with 10^−4^ M phenylephrine and relaxed with a 10^−2^ M acetylcholine to test the endothelial integrity. After the washing and stabilization, the reactivity protocol was initiated, where a dose-response relaxation curve was obtained using increasing doses of acetylcholine (after pre-constriction with 10^−4^ M phenylephrine), and a dose-response contraction curve was obtained using increasing doses phenylephrine (10^−10^ to 10^−4^). The role of ROS in contractile responses was evaluated by incubation with the antioxidant enzymes superoxide dismutase (150 U/mL) and catalase (1000 U/mL) and the NADPH oxidase inhibitor apocynin (30 μM) for 30 minutes before the dose-response curve.

### MI extension

The hearts were divided into the apex, medial ring (approximately 3 mm), and base. The medial ring was stored in a histology cassette for baths in xylol and ethanol. Samples were embedded in histological paraffin at 60 °C for microtomy, with cross-sections of 6 μm thickness, and mounted on glass slides. The histological sections were stained with Picrosirius, and the images were digitized using a scanner for subsequent evaluation using the ImageJ software (National Institute of Health, USA).

### Western blotting

Protein expression in the thoracic aorta samples was analyzed by western blotting. The samples were homogenized in lysis buffer containing (in mmol/L) 150 NaCl, 50 Tris-HCl, 5 EDTA-2Na, and 1 MgCl_2_ plus protease inhibitor (Sigma Fast, Sigma). The protein concentration was determined by the Lowry method [18] using bovine serum albumin as the standard. Equal amounts of protein (50 μg) were electrophoresed (2:30 hours at 80V) in 10% polyacrylamide gel (SDS-PAGE). Next, the proteins were transferred to polyvinylidene fluoride (PVDF) membranes for a period of 1:40–2:30 hours at 60V in a wet blotting system. After the transfer, the membrane was blocked with TBS-T + 5% milk for 2:30 hours and then washed with TBS-T for 5 minutes six times. The membranes were incubated for 4 hours with monoclonal anti-mouse antibodies to catalase (CAT; 1:2000; Sigma, USA) and anti-rabbit polyclonal antibodies to superoxide dismutase (SOD-2; 1:500; Sigma, USA), and gp91phox (1:1000; BD, New Jersey, USA). The membranes were washed with TBS-T and then incubated with the anti-mouse IgG secondary antibodies (1:3000, Abcam Inc., Cambridge, MA, USA) or an anti-rabbit antibody (1:7000; Santa Cruz Biotechnology, CA, USA). The bands were visualized using an NBT/BCIP system (Invitrogen Corporation, CA, USA) and analyzed by the ImageJ software (National Institute of Health, USA). β-actin expression was assessed using a monoclonal anti-mouse antibody to β-actin (1:5000; Sigma Chemical Co., St. Louis, USA). The results were calculated using the ratio of the density of the proteins of interest corrected for the intensity of the protein used as the control (β-actin).

### Fluorescence produced by the oxidation of dihydroethidium (DHE)

To determine the influence of the treatments on the production of superoxide, the fluorescence produced by the oxidation of DHE was used. This method allows the *in situ* analysis of superoxide production. The aortic segments were isolated and kept for 1 hour in Krebs-Henseleit solution with 30% sucrose. These segments were then frozen in a suitable medium (freezing medium, Tissue Tek-OCT). The samples were stored at -80 °C until the day of the experiment.

The aortic segments were sliced in a cryostat into 8-μm thick rings. After this procedure, the slides containing the slices were washed and subsequently incubated with Krebs HEPES (per 100 mL Krebs: 29.4 mg CaCl_2_, 759 mg NaCl, 41.7 mg KCl, 4.9 mg MgCl_2_, 197.8 mg HEPES, and 198.2 mg glucose) for 30 minutes in a humid chamber at 37 °C. After 30 minutes, the Krebs solution was drained, and the excess was dried. Then, the sample was incubated with DHE for 2 hours in a closed chamber incubator at 37 °C.

The emitted luminescence was visualized with a confocal fluorescence microscope (Leica 2500 DM) and a photographic camera (NIKON Digital Sight DS-U2) with a fluorescence filter for DHE (red). The ethidium attached to the nucleus of the cell was visualized with λexc = 585 nm and detected with λem = 600-700nm. The images were quantified using the Image-Pro Plus software (Media Cybernetics, Inc., USA). The signal intensity was analyzed throughout the entire circumference of the three vessel sections by the same investigator.

### *In situ* nitric oxide detection (DAF-2)

The detection of NO production was made using 4,5-diaminofluorescein diacetate (DAF-2) in aortic sections, as described previously [19]. After dissection, aorta samples were embedded and frozen in a freezing medium (Tissue Tek-OCT) and then was stored at -80° C until analysis. Transverse sections (10 μm) were sliced by a cryostat and equilibrated for 30 min at 37° C in phosphate buffer (0.1 M) containing CaCl_2_ (0.45 mM). Then, the sections were incubated with DAF-2 (8 μM) diluted in phosphate buffer (0.1 M) containing CaCl_2_ (0.45 mM) in a humidified chamber protected of the light. The digital images were collected with a confocal fluorescence microscope (Leica 2500 DM) and a photographic camera (NIKON Digital Sight DS-U2). The fluorescence density was quantified by the ImageJ software (National Institutes of Health, USA) and the results were obtained by the mean from three sections of the same animal made by the same investigator.

### Statistical analysis

Data were expressed as the mean ± standard error of the mean (SEM). The histological analysis parameters were compared between the infarcted groups using the unpaired Student’s *t*-test. The vascular reactivity in aortic rings was analyzed by the *two-way* Analysis of Variance (ANOVA). The other data were analyzed by the *one-way* ANOVA followed by the Fisher’s *post-hoc* test. The significance level adopted was p <0.05.

## Results

### MI area

**Fig 1:**
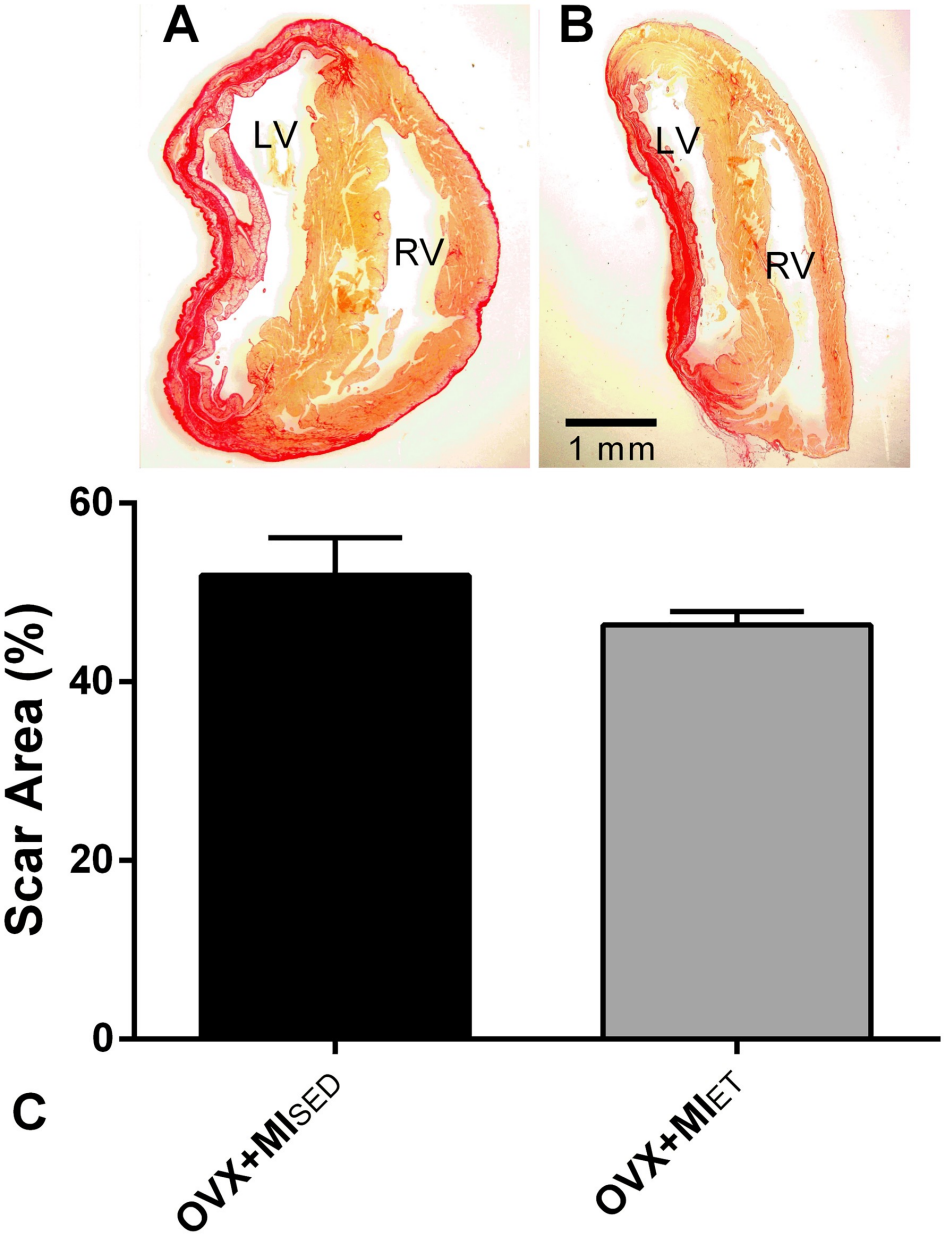
Histological analysis of myocardial infarction area. Area of infarct extension stained with Picrosirius (A and B). Representative graph of the statistical analysis of infarct extension (C). Data are expressed as mean ± SEM (n = 5 and 4, respectively). Unpaired Student´s t-test was used to compare the groups.

The hearts of the OVX+MI_ET_ and OVX+MI_SED_ groups were subjected to histology to assess the size of the infarct area. As shown in Fig 1, the infarcted group that did not perform any ET had a mean infarct area of 51,91%, while the infarcted group subjected to ET showed an area of 48,98%. It was not detected differences between the groups (p> 0.05).

### Estrogen status and body weight

As demonstrated in Table 1, all ovariectomized groups had significantly lower uterine weight compared to the control group, even when corrected by the body weight (p< 0.05). This result was expected since OVX tends to cause atrophy of the uterus and, consequently, a reduction in the uterine weight. This result reflects the effectiveness of the surgery and the estrogen condition intended in the study. In addition, the ovariectomized animals presented an increase in the final body weight compared to that of the control group (p< 0.05).

**Table 1:** Body and organ weights.

	Control	OVX+SHAM_SED_	OVX+SHAM_ET_	OVX+MI_SED_	OVX+MI_ET_
**Final body weight (g)**	276 ± 9,36	323 ± 16,59 *	311 ± 10,44 *	306 ± 11,08 *	305 ± 6,00 *
**Uterus weight (mg)**	794 ± 76	149 ± 36 *	159 ± 37 *	90 ± 8 *	110 ± 13 *
**UW/BW (mg/g)**	3,07 ± 0,345	0,377 ± 0,128 *	0,392 ± 0,033 *	0,300 ± 0,030 *	0,262 ± 0,022 *
**Wet lung (mg)**	1318 ± 71	1426 ± 113	1346 ± 60	1716 ± 166	1851 ± 180 *#$
**WL/BW (mg/g)**	4,48 ± 0,573	4,04 ± 0,241	4,39 ± 0,281	5,26 ± 0,531	5,39 ± 0,357 #
**Dry lung (mg)**	309 ± 26	311 ± 17	314 ± 18	406 ± 41 *	414 ± 30 *#$
**DL/BW (mg/g)**	1,13 ± 0,103	0,97 ± 0,058	1,00 ± 0,048	1,30 ± 0,115	1,38 ± 0,112 #$
**Lung water content (%)**	76 ± 0,706	76 ± 0,719	76 ± 0,963	74 ± 1,491	76 ± 0,720
**Retroperitoneal fat (g)**	7,62 ± 1,05	10,30 ± 1,90	7,31 ± 1,52	5,85 ± 0,66 #	4,60 ± 0,51 *#$
**Parametrial fat (g)**	7,72 ± 0,63	7,92 ± 1,16	6,89 ± 0,83	7,52 ± 1,28	5,52 ± 0,53 *
**Mesenteric fat (g)**	3,79 ± 0,31	5,42 ± 0,76 *	4,27 ± 0,58	4,48 ± 0,46	3,79 ± 0,32 #
**Total fat (g)**	19,12 ± 1,54	23,66 ± 3,53	18,48 ± 2,40	17,86 ± 1,81	13,74 ± 1,22 *#

Data are presented as mean ± SEM. One-way ANOVA followed by the Fisher´s *post-hoc* test. UW, Uterus weight; BW, Body weight; WL, Wet lung; DL, Dry lung.

*p< 0.05 *vs* Control group;

^#^p< 0.05 *vs* OVX+SHAM_SED_;

^$^p< 0.05 *vs* OVX+SHAM_ET_.

### Vascular reactivity

**Fig 2:**
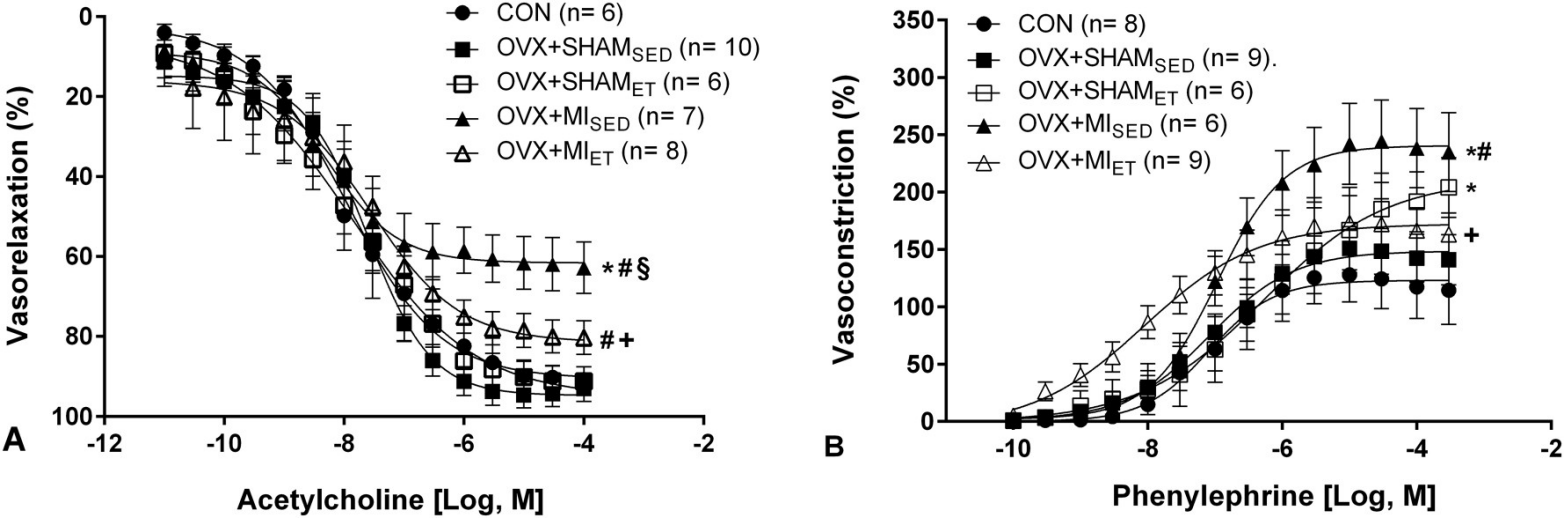
Concentration-response curves to acetylcholine (A) and phenylephrine (B) in isolated aortic rings of Wistar rats. Data are expressed as mean ± standard error of the mean (SEM). *Two-way* ANOVA followed by the Fisher *post-hoc* test for multiple comparisons. *p <0.05 *vs* control; ^#^p <0.05 *vs* OVX+SHAM_SED_;. ^§^p <0.05 *vs* OVX+SHAM_ET_; ^+^p <0.05 *vs* OVX+MI_SED_.

Animals subjected to MI showed greater vessel contractility to phenylephrine (Fig 2A) and lower endothelium-mediated vasodilation to acetylcholine (Fig 2B) compared to those in the control and OVX+SHAM_SED_ groups (p< 0.05). However, ET in MI rats was able to prevent the damage to both vasoconstriction and vasodilation reactivity.

**Fig 3:**
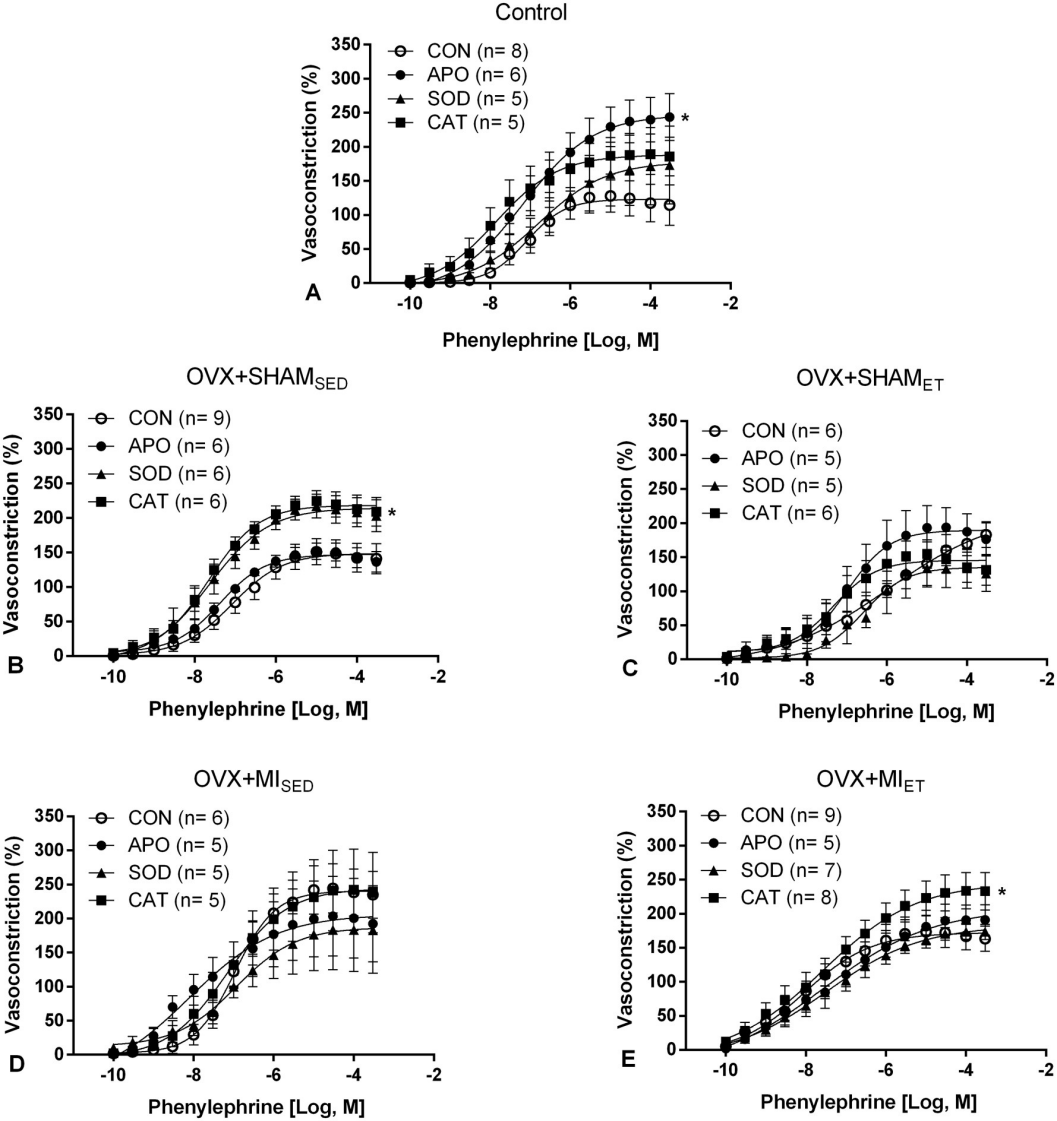
Concentration-response curves to phenylephrine after incubation with apocynin, SOD, and catalase in isolated aortic rings. Data are expressed as mean ± standard error of the mean (SEM); *Two-way* ANOVA was used followed by the Dunnet *post-hoc* test for comparisons with the control group curve *p <0.05 *vs* control.

To evaluate the role of the antioxidant system, the aortic rings were incubated with superoxide dismutase, catalase, and apocynin, and the concentration-response curve was obtained with phenylephrine as described above. The NADPH oxidase inhibitor, Apocynin, promoted effects on vessel contraction of larger magnitude in the control animals, while the other enzymes did not alter the percentage of contraction (Fig 3A). SOD and catalase increased the contractility to phenylephrine in the OVX+SHAM_SED_ group (p< 0.05 *vs* Control). This effect was not observed with the other studied enzymes (Fig 3B). None of the enzymes promoted differences in the contractile responses in the OVX+SHAM_ET_ group (Fig 3C), as well as in the OVX+MI_SED_ group (Fig 3D). However, it was observed increased contractility in vessels containing catalase (p< 0.05) from the OVX+MI_ET_ animals (Fig 3E).

### Protein expression

**Fig 4:**
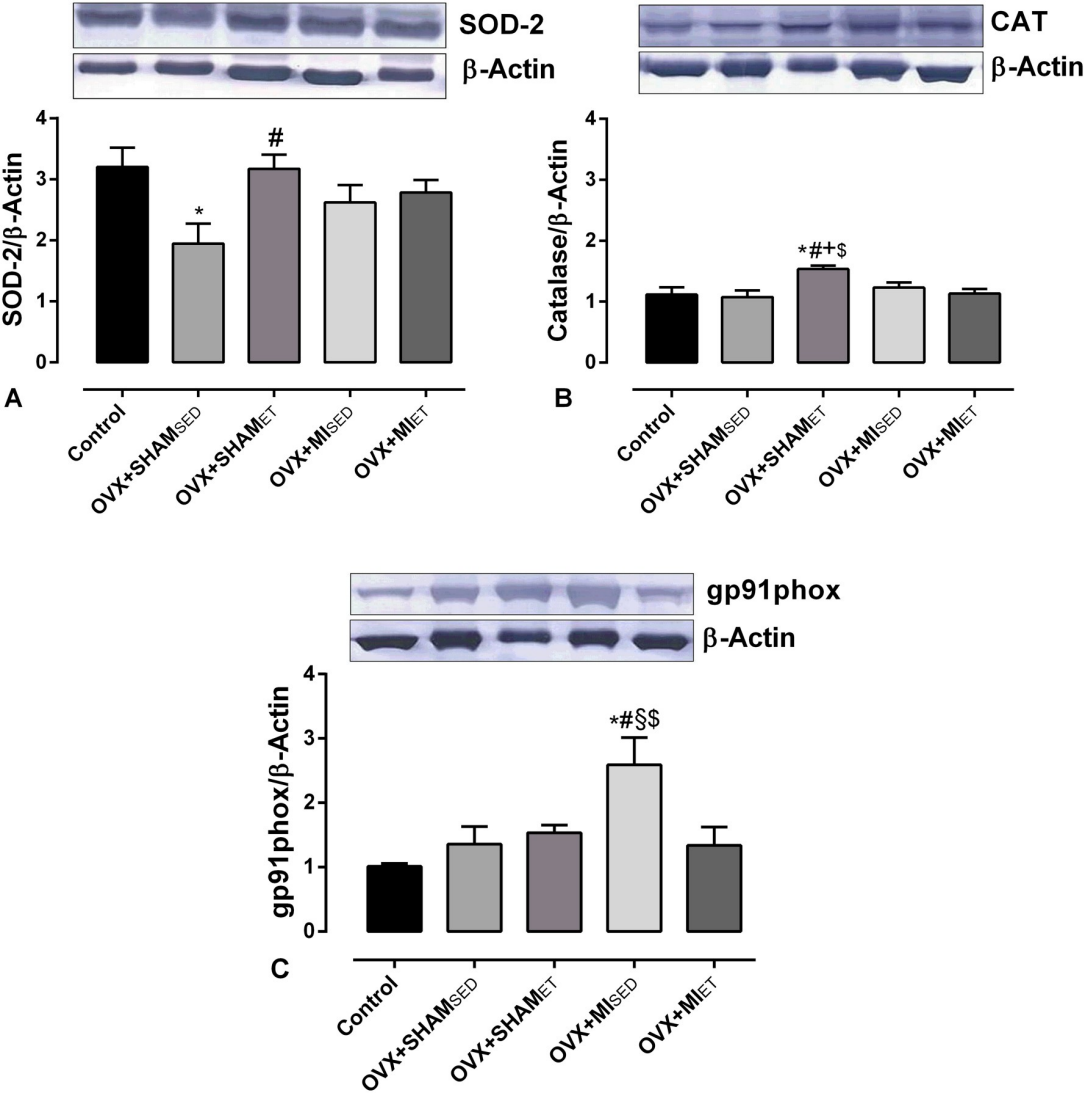
Protein expression of the enzymes SOD-2, Catalase, and gp91phox. Data are expressed as mean ± standard error of the mean (SEM). *One-way* ANOVA followed by the Fisher *post-hoc* test for multiple comparisons. *p <0.05 *vs* Control; ^#^p <0.05 *vs* OVX+SHAM_SED_; ^§^p <0.05 *vs* OVX+SHAM_ET_; ^+^p <0.05 *vs* OVX+MI_SED_; ^$^p <0.05 *vs* OVX+MI_ET_.

In the analysis of the antioxidant enzymes, Fig 4A shows that the superoxide dismutase expression was significantly higher in the control and OVX+MI_ET_ groups (p< 0.05) compared to the OVX+SHAM_SED_ group. In contrast, the catalase protein expression was higher in the OVX+SHAM_ET_ group compared with all other groups (p< 0.05), as shown in Fig 4B.

The expression of the NADPH oxidase subunit gp91phox (Fig 4C) showed that the reduction in ovarian hormones did not affect its expression in the aortic vessel. On the other hand, MI promoted an increase in its expression in the OVX+MI_SED_ group compared with other groups (p< 0.05). However, ET was able to prevent the increase in expression in the MI rats submitted to ET.

### Superoxide and nitric oxide production

**Fig 5:**
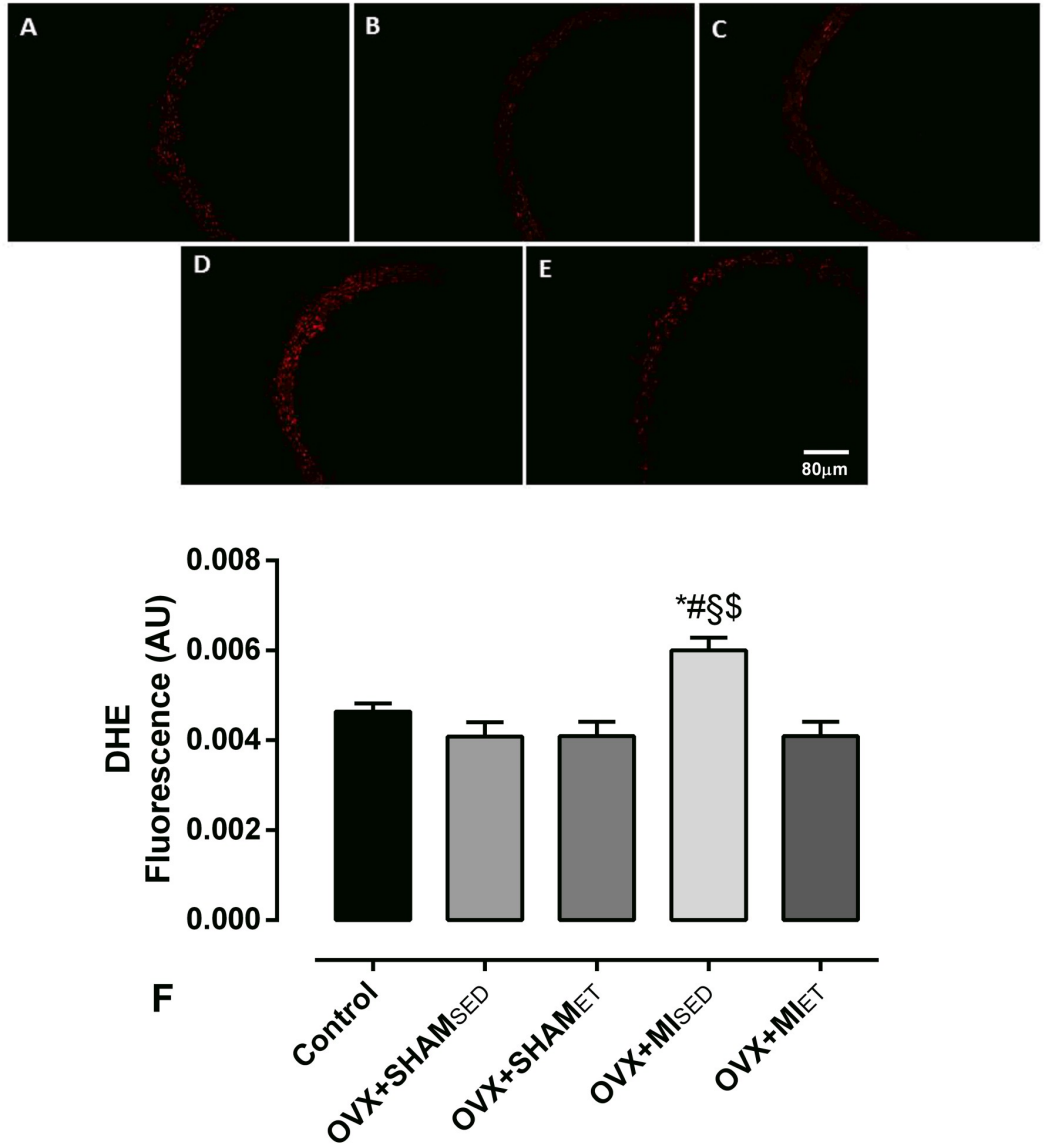
Evaluation of superoxide production *"in situ"* by micrographs depicting the fluorescence emitted by dihydroethidium in segments of the aorta vessels. Data are expressed as mean ± standard error of the mean (SEM). *One-way* ANOVA followed by the Fisher *post-hoc* test for multiple comparisons. *p <0.05 *vs* Control; ^#^p <0.05 *vs* OVX+SHAM_SED_; ^§^p <0.05 *vs* OVX+SHAM_ET_; ^$^p <0.05 *vs* OVX+MI_ET_.

**Fig 6:**
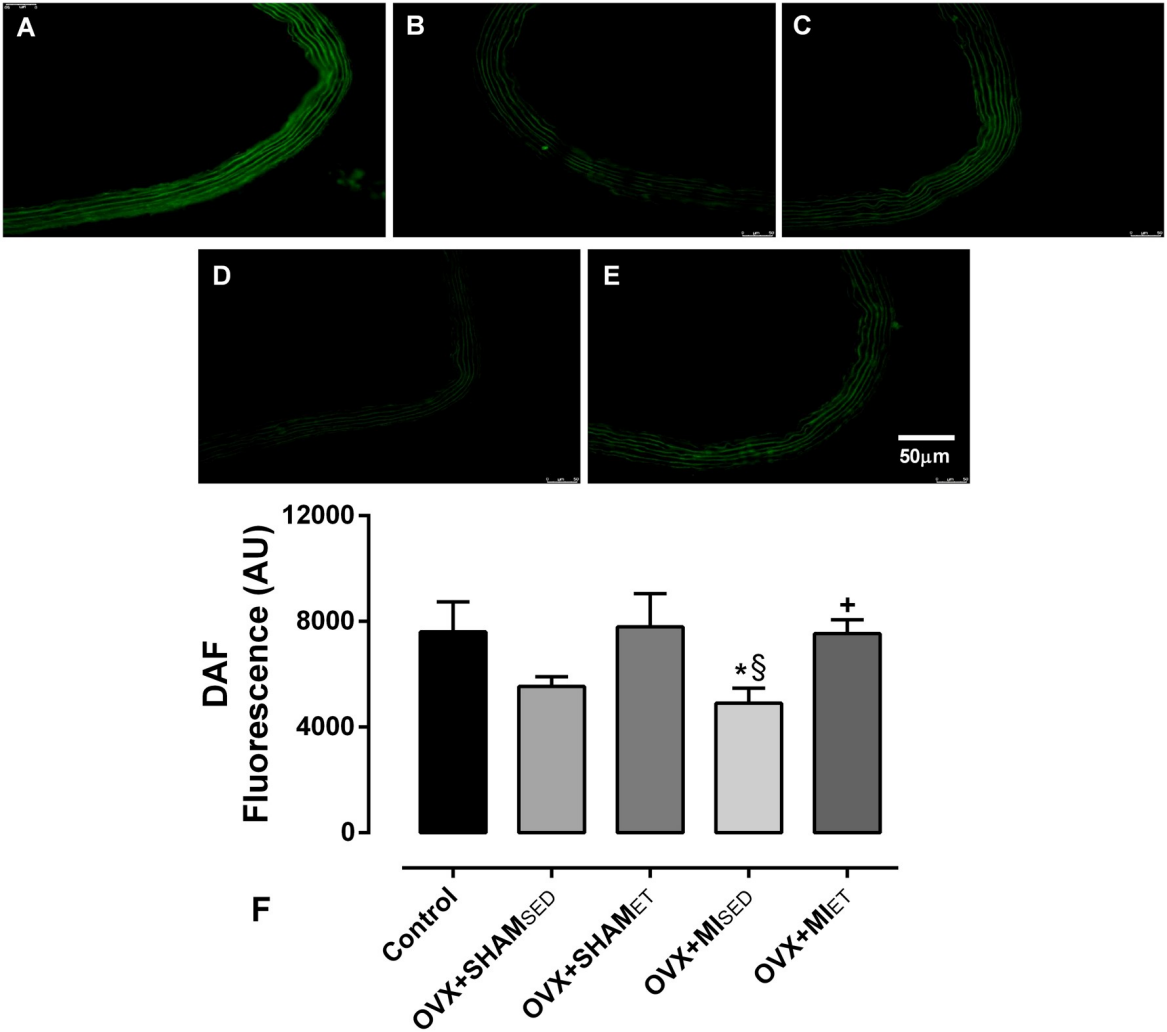
Evaluation of nitric oxide production *"in situ"* by micrographs depicting the fluorescence emitted by 4,5-diaminofluorescein diacetate in segments of the aorta vessels. Data are expressed as mean ± standard error of the mean (SEM). *One-way* ANOVA followed by the Fisher *post-hoc* test for multiple comparisons. *p <0.05 *vs* Control; ^§^p <0.05 *vs* OVX+SHAM_ET_; ^+^p <0.05 *vs* OVX+MI_SED_.

The superoxide production was significantly higher in the OVX+MI_SED_ animals compared to the other groups (Fig 5). However, the ET performed by infarcted animals was able to reduce the excessive production of superoxide, as indicated by the significantly lower superoxide production in the OVX+MI_ET_ group compared to that in the OVX+MI_SED_ group. The *in situ* analysis of NO (Fig 6) showed that MI reduces its production (p< 0.05 *vs* Control and OVX+SHAM_ET_). Nevertheless, ET was able to prevent the NO reduction in the MI rats because the NO production was significantly higher when compared with its respective sedentary group (p< 0.05).

## Discussion

The aim of the present study was to evaluate the effects of ET on the vascular reactivity and the role of ROS in ovariectomized and infarcted female rats. Therefore, in the vascular reactivity analysis, MI impaired vessel function, exhibited by decreased relaxation and increased contractility. MI is known to trigger the activation of neurohumoral mechanisms, such as the renin-angiotensin-aldosterone system and greater activation of the sympathetic nervous system, which leads to major changes in vessel reactivity [20,21], caused by an imbalance between the release of vasoconstrictor and vasodilatory factors [22]. In accordance, Bianchi et al., 2006 [4] showed that MI increases the vasoconstrictor responses to phenylephrine in aortic rings of ovariectomized rats. Furthermore, acute MI lead to an inflammatory process in which pro-inflammatory cytokines, such as TNF-α, IL-6, and IL-1β, have been identified as the most relevant inflammatory mediators. Sustained high levels of cytokines may trigger endothelial dysfunction [23,24]. Similar studies have shown impairments in vascular reactivity caused by MI in coronary vessels and in mesenteric arteries [25,26]. In aorta arteries, it was reported that MI also has led to impairments in the reactivity only 3 days after the MI induction [27]. In agreement, our results suggest that the changes in the contractility pattern in the infarcted group were mainly promoted by the infarction, and not by the OVX, as the sham group did not differ from the control group.

The main finding of the study in the vascular reactivity analyses was the improvement in the OVX+MI_ET_ group compared to the OVX+MI_SED_ group. In addition, ET was able to prevent the reduction in the NO production in the vessels of the infarcted rats, as verified by the DAF analyses. Therefore, low-to moderate-intensity aerobic ET that began 15 days after MI was sufficient to promote an increase in the relaxation and reduction in contractility, which approached the basal levels that had been altered by the MI. ET has been shown to promote acute and chronic changes in vascular reactivity [28–30]. The increases in pulsatile force and shear stress have been reported to be the main mechanical parameters responsible for the acute changes, and exercise-induced shear stress leads to vasodilation [31]. In turn, several studies have reported that the main observed chronic effect of ET on vascular reactivity is the minimization of the vasoconstrictor effect by vasodilatory influences [31,32]. Such mechanical action induces the activation of endothelial nitric oxide synthase (eNOS), which in turn produces NO. The reduction in the calcium influx into smooth muscle cells that cause contraction and, an increase in the calcium concentration in endothelial cells increasing the phosphorylation of eNOS has been reported to be other important effects related to ET on the vessels [33,34].

Investigations of the effects of ET on the vascular function are quite heterogeneous and cover factors such as gender, the existence or absence of disease, age, and other aspects. Some studies have demonstrated that moderate to high-intensity ET provides greater benefits to cardiovascular and pulmonary functioning than lower intensity activities [35]. On the other hand, other studies reported vascular damage induced by the practice of high-intensity ET, such as a reduction in antioxidant enzymes, increase in contractility, and impairment of the relaxation [36,37].

Our data suggest that the improvement in vascular function occurred through prevention in the increase of gp91phox expression. The OVX+MI_ET_ group showed lower protein expression of gp91phox than the OVX+MI_SED_ group, indicating that the sedentary groups worsen its vascular function mainly because of greater protein expression of this enzyme. Since NADPH oxidase is one of the major sources of the superoxide anion and greater activation of this enzyme results in oxidative stress. This result is confirmed by the DHE analysis, which showed that the superoxide production was significantly higher in the OVX+MI_SED_ group than in the OVX+MI_ET_ group.

Regarding the hypothesis that ET could modulate the antioxidant system, we sought to investigate the effect of the enzymes superoxide dismutase and catalase on vascular reactivity. The control group presented greater responsiveness to phenylephrine in vessels receiving apocynin (an inhibitor of the NADPH oxidase enzyme complex). The superoxide anion is described as a NO scavenger, because when both reacts generates ONOO-, thereby reducing the NO bioavailability [6–10]. Because NO is a vasodilator factor, inhibiting the source of superoxide was expected to decrease the contractile response. However, a contradictory response was observed in the group not subjected to surgical procedures or ET. It is known that the exacerbated production of ROS is detrimental and it can trigger numerous diseases. However, the normal production of the superoxide anion by the NADPH oxidase pathway observed under physiological conditions is important for the maintenance in cell signaling functions [38]. Since these animals were probably not in situations of oxidative stress and instead had low levels of ROS production, blocking the superoxide production was detrimental to the vessel reactivity.

The OVX+SHAM_SED_ group showed that the percentage of contraction was similar between the control and apocynin vessels. Activation of NADPH oxidase in this group might be low since the animals were not subjected to MI. Therefore, the blockade of this enzymatic complex did not promote major changes. The vessels that received the antioxidant enzymes presented a higher degree of contractility, with a significant difference between the vessels that received catalase and the control vessels. This result was not observed in the animals subjected to ET, as no significant differences in the contractile curves of the OVX+SHAM_ET_ animals were observed; however, a contractile behavior similar to that of the sedentary group was maintained, keeping the control and apocynin curves at closer values, in contrast to the antioxidant enzymes. Opposite results were observed among the infarcted animals, in which there was no significant difference between the curves of the OVX+MI_SED_ animals; however, in the OVX+MI_ET_ animals, the vessels that received catalase showed a higher percentage of contraction.

The western blotting data show that OVX promoted a reduction in the protein expression of SOD. However, ET was important for avoiding a reduction in the expression of this antioxidant enzyme, since the OVX+SHAM_ET_ group did not differ from the control group. In addition, the OVX+MI_ET_ group had higher expression of this enzyme than the sedentary sham group. In contrast, the catalase expression was significantly higher in the OVX+SHAM_ET_ animals than in the control and OVX+SHAM_SED_ groups, demonstrating the efficiency of ET for this group. However, among the infarcted animals, ET did not promote differences in catalase expression.

## Conclusions

In conclusion, MI impairs the endothelium-dependent and endothelium-independent vascular reactivity in the aorta of ovariectomized rats, and the increase in oxidative stress through the higher expression of gp91phox contribute to these results. In addition, ET improves vascular reactivity in MI rats by preventing the increase in the expression of gp91phox and the decrease in the enzymatic antioxidant system, resulting in a normal ROS production and the maintenance of NO bioavailability. Thus, ET can be an effective therapeutic strategy for improving the MI-induced vascular alterations in the estrogen deficiency condition.

## Abbreviations

CAT: Catalase
CVD: Cardiovascular diseases
DHE: Dihydroethidium
eNOS: Endothelial nitric oxide synthase
ET: Exercise training
gp91phox: Glycoprotein 91 phagocyte oxidase
MI: Myocardial infarction
NADPH: Nicotinamide adenine dinucleotide phosphate
NO: Nitric oxide
O_2_: Oxygen
ONOO^-^: Peroxynitrite
OVX: Ovariectomy
ROS: Reactive oxygen species
SOD: Superoxide dismutase
TNF-α: Tumor necrosis factor alpha
